# Protective Effect of Hydroxytyrosol on LPS-Induced Inflammation and Oxidative Stress in Bovine Endometrial Epithelial Cell Line

**DOI:** 10.3390/vetsci7040161

**Published:** 2020-10-23

**Authors:** Enrico Gugliandolo, Roberta Fusco, Patrizia Licata, Alessio Filippo Peritore, Ramona D’amico, Marika Cordaro, Rosalba Siracusa, Salvatore Cuzzocrea, Rosalia Crupi

**Affiliations:** 1 Department of Chemical, Biological, Pharmaceutical and Environmental Science, University of Messina, 98166 Messina, Italy; egugliandolo@unime.it (E.G.); rfusco@unime.it (R.F.); aperitore@unime.it (A.F.P.); rdamico@unime.it (R.D.); cordarom@unime.it (M.C.); rsiracusa@unime.it (R.S.); 2 Department of Veterinary Science, University of Messina, 98168 Messina, Italy; plicata@unime.it (P.L.); rcrupi@unime.it (R.C.)

**Keywords:** endometrial epithelial cell, bovine endometritis, hydroxytyrosol

## Abstract

Bovine endometritis is a serious pathogen-induced infectious disease that affects the physiological processes of estrus, pregnancy and the postpartum condition. The inflamed endometrium responds by activating an inflammatory intracellular signaling cascade that leads to increased expression of proinflammatory cytokines and reactive oxygen species (ROS). Oxidative stress is closely related to several pathological conditions in perinatal dairy cows and play a key role in tissue damage. Hydroxytyrosol (HT), a natural phenolic alcohol with a strong antioxidant activity, displayed a wide range of biological effect. The aim of this study was to evaluate the protective effects of HT in an in vitro model of lipopolysaccharide (LPS)-induced inflammation in bovine uterine endometrial cells. Our results showed that HT had a significant protective effect in LPS-induced inflammation and oxidative stress. HT was also able to increase the capacity of endogenous antioxidant systems through the up-regulation of the NRF2 pathway. Furthermore, HT restored the tight junction protein expressions. In conclusion, our results showed the protective effects of HT in LPS-stimulated BEND cells. Therefore, the results of this study suggest an important protective role of HT in the treatment and prevention of uterine pathologies in dairy cows.

## 1. Introduction

Bovine endometritis is a serious pathogen-induced infectious disease that affects the physiological processes of estrus, pregnancy and the postpartum condition. During pregnancy, uterine inflammation activates the inflammatory responses involved in maternal-fetal crosstalk and may cause premature birth [1,2,3]. It could result in decreased milk production and long-term infertility. Currently, bovine endometritis represents a serious worldwide economic problem for the cattle industry, as it is highly associated with reduced reproductive activity [4,5]. This is also translated into an increase in the costs for the farm and an increase in the cost per liter of milk. Thus, investigating the pathways and processes involved in endometrial inflammation are important steps for understanding the events that occur in the presence of pathogens and may result in embryo loss, pregnancy failure and infertility [6,7]. Trueperella pyogenes and Escherichia coli are often associated with the uterine infection [8]. Pathogenic microorganisms that attack the reproductive tract are firstly recognized by the cells of the innate immune system within the binding of pattern-recognition receptors to the pathogen-associated molecular patterns (PAMPs) [9]. PAMPs including lipopolysaccharides (LPS), constituents of the cell wall of Gram-negative bacteria, strongly activate the immune system and trigger the inflammatory answer [10,11,12]. The inflamed endometrium responds by activating an inflammatory intracellular signaling cascade that leads to increased expression of proinflammatory cytokines and reactive oxygen species (ROS) [13,14,15]. This oxidative stress damages different biomolecules including proteins, nucleic acids and lipids [16]. Additionally, it leads to the activation of transcription factors that propagate the inflammation. Nevertheless, several examples of evidence indicate that prolonged proinflammatory insults and oxidative stress can negatively affect uterine normal function and block embryonic development [1,2,3]. Despite different studies about bovine endometritis, its treatment did not advance during these decades, and it still trusts broad spectrum antibiotics, which leads to a decrease in immunity and an increase in bacterial resistance, as well as residues of drugs in meat and milk. Hence, new effective treatments against bovine endometritis should be investigated. Hydroxytyrosol (3,4-dihydroxyphenylethanol, HT), a biological antioxidant, has been reported to improve cellular defenses against oxidative injury. It is one of major polyphenol constituent of the plant Olea europaea L., which belongs to the Oleaceae family. HT represents the 80% of the total phenolic fractions of extra virgin olive oil [17]. Elevated HT concentration has also been detected in olive mill wastewater and leaf extract [18]. In particular, numerous beneficial activities have been attributed to HT and its application has been described in different diseases [18,19,20,21,22,23,24]. From the molecular point of view, HT enhanced the Nrf2 pathway [25,26] and ameliorated the antioxidant activity of several enzymes such as superoxide dismutase (SOD), glutathione peroxidase (GPx) and catalase (CAT) [27]. A recent study of our laboratory showed the positive effects of HT on oxidative stress and inflammatory response in bovine mammary alveolar (MAC-T) cells, proposing it as therapeutic strategy for the management of bovine mastitis. Studying large animal systems may be challenging due to the complexities of multi-cell interactions [28,29,30,31]. Therefore, the aim of this study was to evaluate the protective effects of HT in an in vitro model of LPS-induced inflammation in bovine uterine endometrial cells.

## 2. Materials and Methods

### 2.1. Cell Culture

The BEND cells were purchased from American Type Culture Collection (ATCC; Manassas, VA, USA). They were derived from the uterine endometrium of on Day 14 of the estrous cycle [32]. Cells were cultured in DMEM/F12 medium containing 10% (*v/v*) FBS and 0.5% (*v/v*) penicillin streptomycin (Sigma, Italy) and incubated at 37 °C in a humidified atmosphere of 5% CO_2_. Every 48 h, fresh medium was supplied. At 80–90% confluency cells were split using 0.25% trypsin solution. The cells were cultured in cell flasks, their morphology was checked daily and prepared for the following experiments. All experiments were performed between the 3rd and 4th passage.

### 2.2. Cell Treatment

BEND cells (2 × 10^6^ cells/mL) were seeded in six-well plates (2 mL/well) at confluent (80–90%), were treated with HT (10 μM and 25 μM) (Sigma-Aldrich, Milano, Italy). One hour after HT treatment, cells were stimulated with LPS 1 μg/mL (Escherichia coli, Sigma-Aldrich, Milano, Italy) for 6 h, as already described [33], and each treatment was replicated 3 times. LPS concentrations were chosen based on previous studies by others using endometrial epithelial cells [33].

### 2.3. Cell Viability Assay

The possible toxic effect of HT on BEND cells was evaluated by methyl thiazolyl tetrazolium (MTT) assay as already shown [34]. Briefly, a suitable amount of cell suspension (2 × 10^6^ cells/mL) was inoculated onto a 96-well plate (100 μL/well) and pre-cultured in a 37 °C, 5% CO2 incubator, then cells were incubated with HT at 10, 25, 50, 100, and 250 μM, for 24 h, followed by the MTT treatment (10 μL of 0.5 mg/mL) for 4 h. The optical density at 550 nm was measured using a microplate reader and used to calculate the cell viability.

### 2.4. ELISA

Secretions of TNF-α and IL-6 were measured using commercial ELISA kits from Cusabio (Houston, TX, USA). Briefly, cell supernatants were centrifuged for 10’ at 3000 rpm. The supernatants were collected and used for ELISA kits according to the manufacturer’s protocol. Briefly 50 μL of Standard (to create a 4-point standard curve) or Sample was added for each well, and subsequently 50 μL of HRP-conjugate and antibody were added and incubated for 1 h. Then the plate was washed three times and 50 μL of substrate (A and B) were added for 15’, to which finally was added 50 μL of stop solution, and absorbance at 450 nm was recorded [35]. The assay was performed in duplicate for each standard and sample, then the duplicate readings were averaged, and the average optical density of Blank was subtracted. The results were obtained by creating (with GraphPad Prism v. 8) a standard curve, generating a four-parameter logistic curve fit (4-PL) and then interpolating the OD of the samples [36]. The results are expressed as concentrations ng/mL. For both TNF-α and IL6 tests used Intra-assay Precision and Inter-assay Precision were CV < 15%.

### 2.5. Reactive Oxygen Species (ROS) Evaluation

Total cellular ROS were evaluated using the 2′,7′-dichlorodihydrofluorescein diacetate (H2DCFDA) dye. BEND cells were grown to confluence, trypsinized, and then washed twice with washing buffer. Next, cells were incubated with H2DCFDA dye 1 μM at 37 °C for 60’. Then the cells were washed and then kept at room temperature for an additional 30 min to allow for complete dye de-esterification. Then, excitation and emission were monitored at 490 nm and 530 nm, respectively, using a fluorescence plate reader [37]. The levels of increased ROS production were expressed as percentage of the control (nmol/mL).

### 2.6. RNA Extraction-cDNA Synthesis

To evaluate the mRNA expression of target genes, RNA was extracted from BEND cells using RNeasy kit (Qiagen, Italy), for real-time polymerase chain reaction (PCR) analysis. Briefly, samples were lysed, and subsequently ethanol was added for ideal binding conditions. The lysates obtained were loaded into the RNeasy silica membrane. RNA binds into column and all contaminants were washed out. Pure, concentrated RNA was eluted in 50 μL water. RNA was quantified with a spectrophotometer (NanoDrop Lite; Thermo Fisher Scientific, Wilmington, DE, USA). iScript RT-PCR kit (Bio-Rad, Hercules, CA, USA) was used to synthesize first-strand cDNA according to manufacturer’s recommendations. Briefly, the reverse transcription master mix was prepared adding to 1 μg of RNA template the iScript RT Supermix (5× RT supermix with RNase H+ Moloney (gray cap, 25 or 100 reactions) murine leukemia virus (MMLV) reverse transcriptase, RNase inhibitor, dNTPs, oligo(dT), random primers, buffer, MgCl2 and stabilizers) and the nuclease-free water. The complete reaction mix was incubated in a thermal cycler (Priming 5 min at 25 °C, Reverse transcription 20 min at 46 °C, RT inactivation for one minute at 95 °C).

### 2.7. Real-Time PCR

In total, 1  μL of total cDNA was used to perform Real-time PCR analysis with by SYBR Green method on a StepOnePlus Real-Time PCR System (Applied Biosystems, USA). PCR conditions were: initial denaturation at 95 °C for 15 min, followed by 45 cycles of amplification at 95 °C for 20 s and 60 °C for 40 s. Final extension at 60 °C for 60 s and ahold at 4 °C were then performed. Data analysis was performed using the 2^−∆∆Ct ^ method and the results are expressed as fold-changes [38]. GAPDH was used as an internal control for normalizing relative expression levels between samples. For each target gene, besides the biological replicates, three technical replicates were performed. Negative controls using RNA as a template were also included in all runs to test for the possible genomic DNA contamination of the samples.

### 2.8. Statistical Analysis

For each experiment, three or more independent experiments have been performed, and for each experiment five repeat samples were used. The data resulting from all experiments are expressed as means ± SEM. Statistical differences between groups were compared using ANOVA, followed by Tukey’s test. A p-value of less than 0.05 was considered statistically significant. Data are representative of at least three experiments, means ± SEM; * *p* < 0.05 vs. control; ** *p* < 0.01 vs. control; ^#^ *p* < 0.05 vs. LPS; ^##^ *p* < 0.01 vs. LPS; *** *p* < 0.001 vs. control; ^###^ *p* < 0.001 vs. LPS.

## 3. Results

### 3.1. HT Effect on BEND Cells Viability

Firstly, we evaluated any possible toxic effect of HT on the BEND cells by the MTT cell viability assay. We tested crescent concentrations of HT (10, 25, 50, 100, 250 μM). Cells were pre-treated with HT and subsequently incubated with MTT for 4 h. No changes in cells viability were detected at low HT concentration (HT 10 μM 98.6 ± 0.50; HT 25 μM 98.4 ± 0.67; HT 50 μM 97.8 ± 0.86) compared to the control. At 100 (94.6% ± 1.2) and 250 μM (93.3 % ± 1.56) of HT a significant reduction in cell viability was detected (Table 1).

### 3.2. HT Effect on LPS-Induced Inflammatory Response and Oxidative Stress in BEND Cells

LPS stimulation (1 μg/mL) induced the overexpression of the main pro-inflammatory cytokines IL-6 and TNF-α (Table 2), compared to the control (IL6 (4.30 ± 120.8 vs. 1.70 ± 0.1), TNF-α (304.8 ± 7.41 vs. 252.8 ± 10.8)). HT treatment at 10 and 25 μM prevents the increase of IL6 (HT 10 μM: 1.50 ± 0.11 and HT 25 μM: 1.32 ± 0.16) and TNF-α (HT 10 μM: 241.8 ± 5.2 and HT 25 μM: 237 ± 7.36) in a dose-dependent manner (Table 2). Additionally, to test the antioxidant effect of HT pre-treatment on LPS-induced oxidative stress in BEND cells we quantified the levels of intracellular ROS (Table 2). LPS stimulation increased intracellular ROS expression compared to the control (33.8 ± 1.68 vs. 16.6 ± 0.02), while samples pre-treated with HT at 10 and 25 μM showed reduced levels of intracellular ROS (HT 10 μM: 22.4 ± 1.20 and HT 25 μM: 15.6 ± 1.07) (Table 2).

### 3.3. HT Effect on LPS-Induced Nrf2 Pathway

**Figure 1:**
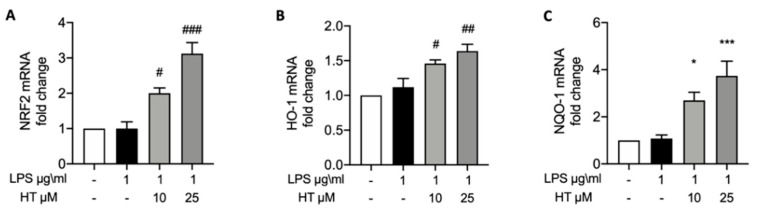
HT pre-treatment on LPS-induced Nrf2 pathway in BEND cell: mRNA levels of Nrf2 (**A**), HO-1 (**B**) and NQO-1 (**C**). * *p* < 0.05 vs control; ^#^ *p* < 0.05 vs. LPS; ^##^ *p* < 0.01 vs. LPS; *** *p* < 0.001 vs. control; ^###^ *p* < 0.001 vs. LPS.

To verify the effect of the HT pre-treatment on the Nrf2 pathway activated by the LPS stimulation RT-PCR were conducted. HT pre-treatment at 10 and 25 μM in a dose-dependent manner up-regulated Nrf2 expression compared to the control (HT 10 μM: 2.00 ± 0.15 and HT 25 μM: 3.12 ± 0.31) (Figure 1A). Nrf2 improves the expression of several antioxidant response genes; in particular, we evaluated Heme oxygenase-1 (HO-1) and NAD(P)H quinone oxidoreductase-1 (NQO-1) expressions by RT-PCR. HT pre-treatment at both concentrations increased HO-1 (Figure 1B) (HT 10 μM: 1.46 ± 0.05 and HT 25 μM: 1.64 ± 0.09) and NQO-1 (Figure 1C) (HT 10 μM: 2.7 ± 0.34 and HT 25 μM: 3.74 ± 0.62).

### 3.4. HT Effect on and Expressions of Tight Junction Proteins

**Figure 2:**
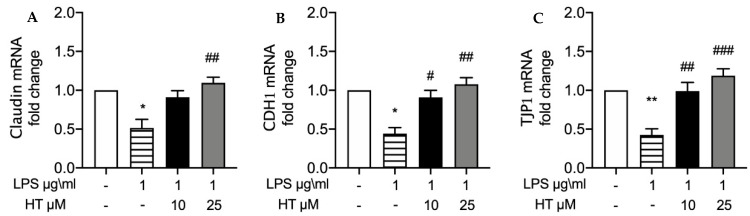
HT pre-treatment on LPS-induced changes in tight junction expressions Nrf2 in BEND cell: mRNA levels of Claudin (**A**), CDH1 (**B**) and TJP1 (**C**). * *p* < 0.05 vs control; ^#^ *p* < 0.05 vs. LPS; ^##^ *p* < 0.01 vs. LPS; ** *p* < 0.01 vs. control; ^###^ *p* < 0.001 vs. LPS.

Tight junctions are involved in tissue protection against pathogen invasion. We employed RT-PCR to investigate the changes in tight junction protein expressions induced by LPS in BEND cells. LPS stimulation significantly reduced Claudin (Figure 2A), CDH1 (Figure 2B) and TJP1 (Figure 2C) mRNA expression compared to the control, (respectively, 0.51 ± 0.11, 0.4 ± 0.07, 0.42 ± 0.07). HT pre-treatment restored in a dose-dependent manner Claudin (HT 10 μM: 0.91 ± 0.08 and HT 25 μM: 1.1 ± 0.07), CDH1 (HT 10 μM: 0.90 ± 0.09 and HT 25 μM: 1.1 ± 0.08) and TJP1 (HT 10 μM: 0.99 ± 0.1 and HT 25 μM: 1.1 ± 0.09) mRNA expressions.

## 4. Discussion

The endometrium is a peculiar mucosa, and in several conditions, it becomes exposed to many bacteria, such as during post-partum periods. Indeed, post-partum periods involve several physiological events such as the expulsion of the placenta, prompt involution of the uterus, which have as their final endpoint a restoration of a receptive endometrium. Many factors influence these events, in particular environmental factors play a key role in increasing the risk of development of post-partum uterine diseases. Uterine disease includes retained placenta, metritis and clinical endometritis, or subclinical endometritis [39]. To date up to 40% of dairy cattle develop post-partum uterine diseases such as endometritis. The bacteria infection of the female genital tract is a severe disease in cattle that leads to infertility, endometrium mucosa damage, and even mortality [40]. *E. Coli* is one of the major pathogens responsible for endometritis [41] and is the main uterine microbial disease in cattle. LPS is the main component of Gram-negative bacteria cell walls. It is well known that LPS induces a strong inflammatory response in both local and systemic tissue through blood circulation. Together with the inflammatory response the increase in oxidative stress is a physiological response against various stimuli such as LPS. Moreover, an imbalance in normal equilibrium with an increase in oxidative stress can occur under conditions in dairy cows [42]. However, increased oxidative stress is closely related to several pathological conditions in perinatal dairy cows and plays a key role in tissue damage [43]. The use of natural compounds is a growing pharmacological approach in the treatment of uterine diseases or prophylaxis. Several studies have shown that the use of natural compounds in combination with other drug therapies is a useful therapeutic strategy [44], as has been seen for example in the case of garlic [45,46] and turmeric [47]. HT is a natural phenolic alcohol with a strong antioxidant activity and has been shown to exert a wide range of biological effects, such as cardioprotective, neuroprotective, and anti-inflammatory effects [48]. Recent evidence has shown the protective effect of HT in bovine mammary epithelial cells, modulating the inflammatory response and reducing the oxidative stress induced by LPS [49,50]. The aim of this study was to evaluate the protective effect of HT on bovine endometrial epithelial cell line (BEND) stimulated with LPS 1 μg/mL. The concentrations of HT used in this study (10 and 25 μM) did not show cytotoxic effects on BEND cells, while a reduction in cell viability was observed for concentrations of 50, 100, and 250 μM; this effect, as seen previously, could be explained by an effect of high HT concentrations on the apoptotic process [51]. Inflammatory response is closely associated with increased oxidative stress, and our results showed a significant increase in ROS levels after LPS stimulation. As previously seen the wide range of biological effect of HT was associated with a strong antioxidant activity, as HT acts as metal-chelator and free radical-scavenger [48]. In particular this strong antioxidant activity, is due to the presence of o-dihydroxyphenyl moiety. In fact, in the presence of free radicals (ROO *) it acts mainly by donating a hydrogen atom. In this way ROO * reacting with HT are transformed into non-reactive compounds. According to the mechanism of action of HT, compared to LPS group we observed a significantly lower ROS level in groups treated with HT 10 and 25 μM, in a dose-dependent manner. We also evaluated the levels of pro-inflammatory cytokines TNF-α and IL-6 as key mediator in inflammatory cascade. Our result showed that BEND cells exposed to LPS (1 μg/mL) respond with a significantly increased level of pro-inflammatory cytokines, such as TNF-α and IL-6. The treatment with HT at 10 and 25 μM showed a significant protective effect in a dose-dependent manner, according to the effect observed on ROS levels. Reduction of IL-6 and TNF-α levels is a key factor for preventing an excessive inflammatory response and consequent complications of the pathology. Furthermore, an increase in oxidative stress is related to an increase in pro-inflammatory cytokine secretion, and in particular oxidative stress-mediated inflammatory responses have long been recognized as important causes of various inflammatory diseases in perinatal dairy cows [43]. The effects of HT in reducing pro-inflammatory cytokine levels observed in this study can be explained by the reduction of LPS-induced oxidative stress. Moreover, the ability of HT in potentiating endogenous antioxidant mechanisms has been demonstrated, and in particular this mechanism of action HT seems to occur through the up-regulation of NRF2. In this study we observed an up-regulation of NRF2 after treatment of HT at 10 and 25 μM in a dose-dependent manner. According to the NRF2 trend we observed, after HT treatment, there is also an increased level of HO-1 and NQO-1 in a dose-dependent manner. Reduction in inflammation and oxidative stress play a key role in maintaining the normal protective barrier. Tight junction proteins are the most important components of the epithelial barrier and play a key role in the maintenance of endometrial homeostasis by restricting the invasion of pathogens, and it has been shown that a reduction of the tight junction proteins occurs in the course of inflammatory pathological processes. Therefore, in this study we decided to evaluate whether the protective effects of HT on inflammation and oxidative stress are translated to a protective effect on the integrity of the mucosa. In particular, we evaluated the expression levels of the tight junction proteins claudin, CDH1 and TJP1. In fact, these tight junctions play a key role in mucosal barrier integrity, and, in particular, this homeostasis is regulated on one side by stimuli on the mucosa wall, such as the presence of bacteria pathogenic, and on the other side they are regulated by immune cells and the levels of cytokines. Immune cells and in particular T cells, play a key role in regulatory function and maintain tight junctions, blocking a major proinflammatory response while maintaining an anti-inflammatory environment. Inflammatory stimuli and cell damage results in a down regulation of the tight junction, as this is a fundamental step for the initiation and progression of pathological processes in the endometrial epithelium [52]. Our results show that in BEND cells exposed to LPS there was a significant reduction of the tight junction proteins claudin, CDH1, and TJP1. In accordance with the reduction of oxidative stress markers and pro-inflammatory cytokines observed above, we observed that treatment with HT at concentrations of 10 and 25 μM had a significant protective effect of the tight junction proteins claudin, CDH1, and TJP1.

## 5. Conclusions

Our results showed that HT had a significant protective effect in LPS-induced inflammation and oxidative stress. HT was also able to increase the capacity of endogenous antioxidant systems through the up-regulation of the NRF2 pathway. Furthermore, these effects also produced a significant protective effect on of the tight junction proteins. Therefore, the results of this study suggest a possible positive outlook, encouraging future clinical animal studies.

## Figures and Tables

**Table 1:** Effects of HT on BEND cells viability.

	LPS -/HT -
**Ctrl**	100 ± 0
**HT 10 μM**	98.6 ± 0.50
**HT 25 μM**	98.4± 0.67
**HT 50 μM**	97.8 ± 0.86
**HT 100 μM**	94.6 ± 1.20^#^
**HT 250 μM**	93.6 ± 1.56^##^

^#^*p* < 0.05 vs. LPS; ^##^ *p* < 0.01 vs. LPS.

**Table 2:** Effects of HT on oxidative stress and inflammation in BEND cells stimulated with lipopolysaccharides (LPS).

	LPS -/HT -	LPS +/HT-	LPS +/HT 10 μM	LPS +/HT 25 μM
**ROS**	16.6 ± 0.02	33.8 ± 1.68 ***	22.4 ± 1.20 ^##^	15.6 ± 1.07 ^###^
**TNF-α**	252.8 ± 10.8	304.8 ± 7.41 **	241.8 ± 5.2 ^#^	237 ± 7.36 ^###^
**IL-6**	1.70 ± 0.1	4.30 ± 120.8 **	1.50 ± 0.11 ^##^	1.32 ± 0.16 ^###^

ROS, reactive oxygen species; TNF-α, tumor necrosis factor α; IL-6, interleukin 6. ** *p* < 0.01 vs control; ^#^ *p* < 0.05 vs. LPS; ^##^ *p* < 0.01 vs. LPS; *** *p* < 0.001 vs. control; ^###^ *p* < 0.001 vs. LPS.
